# Assessment of Myocardial Fibrosis in Mice Using a T2*-Weighted 3D Radial Magnetic Resonance Imaging Sequence

**DOI:** 10.1371/journal.pone.0129899

**Published:** 2015-06-26

**Authors:** Bastiaan J. van Nierop, Noortje A. M. Bax, Jules L. Nelissen, Fatih Arslan, Abdallah G. Motaal, Larry de Graaf, Jaco J. M. Zwanenburg, Peter R. Luijten, Klaas Nicolay, Gustav J. Strijkers

**Affiliations:** 1 Biomedical NMR, Department of Biomedical Engineering, Eindhoven University of Technology, Eindhoven, The Netherlands; 2 Soft Tissue Biomechanics and Engineering, Department of Biomedical Engineering, Eindhoven University of Technology, Eindhoven, The Netherlands; 3 Department of Cardiology, University Medical Center Utrecht, Utrecht, Netherlands; 4 Laboratory of Experimental Cardiology, University Medical Center Utrecht, Utrecht, Netherlands; 5 Department of Radiology, University Medical Center Utrecht, Utrecht, Netherlands; 6 Biomedical Engineering and Physics, Academic Medical Center, Amsterdam, The Netherlands; Scuola Superiore Sant'Anna, ITALY

## Abstract

**Background:**

Myocardial fibrosis is a common hallmark of many diseases of the heart. Late gadolinium enhanced MRI is a powerful tool to image replacement fibrosis after myocardial infarction (MI). Interstitial fibrosis can be assessed indirectly from an extracellular volume fraction measurement using contrast-enhanced T1 mapping. Detection of short T2* species resulting from fibrotic tissue may provide an attractive non-contrast-enhanced alternative to directly visualize the presence of both replacement and interstitial fibrosis.

**Objective:**

To goal of this paper was to explore the use of a T2*-weighted radial sequence for the visualization of fibrosis in mouse heart.

**Methods:**

C57BL/6 mice were studied with MI (n = 20, replacement fibrosis), transverse aortic constriction (TAC) (n = 18, diffuse fibrosis), and as control (n = 10). 3D center-out radial T2*-weighted images with varying TE were acquired *in vivo* and *ex vivo* (TE = 21 μs-4 ms). *Ex vivo* T2*-weighted signal decay with TE was analyzed using a 3-component model. Subtraction of short- and long-TE images was used to highlight fibrotic tissue with short T2*. The presence of fibrosis was validated using histology and correlated to MRI findings.

**Results:**

Detailed *ex vivo* T2*-weighted signal analysis revealed a fast (T2*_fast_), slow (T2*_slow_) and lipid (T2*_lipid_) pool. T2*_fast_ remained essentially constant. Infarct T2*_slow_ decreased significantly, while a moderate decrease was observed in remote tissue in post-MI hearts and in TAC hearts. T2*_slow_ correlated with the presence of diffuse fibrosis in TAC hearts (r = 0.82, *P* = 0.01). *Ex vivo* and *in vivo* subtraction images depicted a positive contrast in the infarct co-localizing with the scar. Infarct volumes from histology and subtraction images linearly correlated (r = 0.94, *P*<0.001). Region-of-interest analysis in the *in vivo* post-MI and TAC hearts revealed significant T2* shortening due to fibrosis, in agreement with the *ex vivo* results. However, *in vivo* contrast on subtraction images was rather poor, hampering a straightforward visual assessment of the spatial distribution of the fibrotic tissue.

## Introduction

Myocardial fibrosis is the formation of excessive collagen and other extracellular matrix (ECM) components in the interstitium and perivascular regions of the myocardium. This accumulation is a consequence of the disruption of the equilibrium between synthesis and degradation of ECM components [1]. Fibrosis is common to ischemic and non-ischemic heart disease, including myocardial infarction (MI), hypertrophic cardiomyopathy and pressure overload induced hypertrophy [2].

Functional consequences of fibrosis are increased myocardial stiffness, decreased myocardial perfusion and perfusion reserve, and mechano-electrical uncoupling. Together these form a substrate for impaired ventricular relaxation leading to diastolic dysfunction, inappropriate pressure development for systemic perfusion resulting in impaired systolic function, ischemia, and arrhythmia [1,3]. There is increasing awareness for the pivotal role of fibrosis in the progression of cardiac pathology towards heart failure (HF) and its relation to poor clinical outcome [4,5].

Fibrosis is characterized by different subtypes [2]. Non-ischemic heart disease, for example due to hypertension, is often associated with diffuse, interstitial fibrosis, but also with perivascular fibrosis. Diffuse fibrosis is generally progressive in nature. Ischemic heart disease can result in replacement or scarring fibrosis and often has a localized distribution, for example as a result of necrosis after MI.

Novel imaging techniques for the characterization of myocardial fibrosis are highly desired, as they could improve risk stratification and aid in the evaluation of new treatment strategies aiming to reduce fibrosis [6,7]. Multiple magnetic resonance imaging (MRI) techniques offer the possibility to detect myocardial fibrosis. For example, qualitative T1-weighted MRI and quantitative T1-mapping MRI after injection of a Gadolinium (Gd) chelate contrast agent have proven highly successful to detect and characterize myocardial fibrosis [2,8]. However, gadolinium contrast is contraindicated for a large group of patients with renal impairment, and in particular for late gadolinium enhanced (LGE) MRI, measurements are complicated by the need for precise inversion timing and often result in overestimation of infarct size in the early phase [9,10].

Alternatively, quantitative changes in the T2 or T2* relaxation times due to the presence of collagen have been exploited to visualize the presence of fibrosis [11]. For instance, T2 was demonstrated to significantly correlate with the extent of diffuse fibrosis in a mouse model of diabetic cardiomyopathy [12]. Moreover, *ex vivo* T2*-weighted imaging using ultrashort echo time (UTE) MRI with shorter and longer TEs was employed to visualize the collagenous scar in a rat MI model [13]. The latter technique seemed particularly promising as it yielded a high contrast in the infarct scar.

To goal of this paper was to explore the use of a T2*-weighted radial sequence for the visualization of fibrosis in the heart and to improve our understanding of T2* changes resulting from fibrotic tissue. The specific aims of this study were therefore: (**i**) To establish an ECG-triggered T2*-weighted 3D center-out radial sequence facilitating a quantitative myocardial T2* measurement in mice with a TE range from 21 μs to 4 ms; (**ii**) To quantify T2* decay in excised myocardial tissue with replacement and diffuse fibrosis; (**iii**) To study the potential of *in vivo* T2*-weighted MRI for assessing replacement and diffuse fibrosis in MI mice and mice with transverse aortic constriction (TAC), respectively.

## Methods

### Animal model

A total of 48 C57BL/6 mice (male, age 11 weeks, 21–28 grams) were included in this study. Animals were housed under standard laboratory conditions with a 12 h light/dark cycle and were maintained on a standard diet with access to water *ad libitum*. All animal experiments were performed according to the Directive 2010/63/EU of the European Parliament and approved by the Animal Care and Use Committee of Maastricht University.

Mice were randomly separated in a control group (n = 8), a group that underwent a permanent occlusion of the left anterior descending (LAD) coronary artery (n = 20) to induce MI and a group that underwent TAC to induce an aortic stenosis, resulting in left ventricular (LV) pressure overload (n = 18) (S1 Methods) [14–16].

### Sequence design

A 9.4 T small animal MRI scanner (Bruker BioSpec, Ettlingen, Germany) was used, equipped with a 72-mm-diameter quadrature transmit coil and a 4-element phased-array receive coil (Bruker). The T2*-weighted sequence consisted of a non slice-selective RF block-pulse (α = 5°, 20 μs, bandwidth = 64 kHz) followed by a 3D center-out radial readout resulting in a minimum echo time (TE) of 21 μs. This sequence may be referred to as a ultra-short echo time (UTE) sequence, but it was also used with longer TE up to 4 ms. Other sequence parameters were: repetition time TR = 6.1 ms, number of averages = 1, field of view = 3x3x3 cm^3^ and matrix size = 128x128x128. To account for eddy-current induced errors and gradient hardware imperfections, actual k-space trajectories and gradient timing were measured and optimized using a phantom [17].

### Study protocol

A group of control (n = 8) mice were randomly selected for *in vivo* MRI measurements at week 6, when the mice were 17 weeks of age. A group of MI mice (n = 18) was measured at 1–2 weeks and TAC mice (n = 12) at 11 weeks after the time point at which they had surgery. After the measurements the anesthetized animals were killed by means of perfusion of the vascular bed with phosphate buffered saline (10 mL, pH 7.4), which was infused via a needle penetrating the apex and exsanguination from the vena cava inferior [18]. Next, heart and lungs were excised and weighed, and tibia length (TL) was determined.

*Ex vivo* MRI measurements were also done in a subset of control (n = 6), MI (n = 15) and TAC hearts (n = 14). To this end, hearts were placed in a cryotube filled with Fomblin (Fens Chemicals, The Netherlands) for susceptibility matching.

### MRI measurements

Mice were anesthetized with 1.5–2.0 vol% isoflurane in 0.4 L/min medical air and placed prone in a dedicated animal cradle. The front paws were placed on ECG electrodes and a balloon pressure sensor was placed on the abdomen. Body temperature was maintained at 36–37°C with a heating pad and monitored with a rectal temperature sensor. The readout of the 3D T2*-weighted sequence was triggered immediately after ECG R-wave detection. Respiratory gating was applied to prevent breathing motion artifacts. To limit the acquisition time to about 14–16 min (depending on mouse heart rate), 3 k-lines were measured after every R-wave and the acquisition matrix was 2x undersampled. A blood-saturation slice (1.5 ms Gauss pulse, α = 90°, slice thickness 3 mm, followed by a 744 μs crusher gradient (185 mT/m) in a short-axis orientation positioned above the LV base provided improved contrast between blood and myocardium. Images were obtained with varying TEs (up to 4 ms). LV function and mass were quantified from two long-axis and a stack of 5–8 short-axis cinematographic (cine) MR images, providing full-heart coverage, with 15–18 frames covering the complete cardiac cycle using an ECG-triggered and respiratory-gated FLASH sequence [19]. To determine infarct location in the post-MI hearts, an LGE scan was performed at the end of the MRI session using 0.5 mmol/kg Gd-DTPA (Bayer HealthCare Pharmaceuticals, The Netherlands) and a self-gated cine FLASH sequence [20].

*Ex vivo* MRI measurements were performed on the freshly excised hearts on the same day. No preservative was used to prevent confounding effects on T2* due to tissue fixation or dehydration [21]. *Ex vivo* T2*-weighted imaging was performed using the same sequence with 34 different TEs between 21 μs and 4 ms, but without the use of a saturation slice. For anatomical reference, a 3D FLASH image was acquired (TR = 15 ms, TE = 5 ms, α = 5°, number of averages = 1, field of view = 3x3x3 cm^3^ and matrix size = 192x192x128).

### Histology

A subset of the control (n = 4), MI (n = 8) and TAC (n = 6) hearts were embedded in paraffin and cut in 5-μm-thick sections. Sections were stained with Picrosirius Red (for collagen) or with Prussian Blue (for iron) according to established histological procedures (S1 Methods).

### Data analysis

The background signal in the *ex vivo* T2*-weighted images was removed using a mask obtained from the 3D FLASH image by applying a signal intensity threshold. Next, long-TE images (4 ms) were subtracted from images with a short-TE (21 μs) to obtain so-called ΔUTE images, suppressing signal with a long T2* and highlighting myocardial tissue with shorter T2* in the infarct area [22]. A signal intensity threshold equal to the mean + 3-times the standard deviation (SD) was applied to a stack of ten remote tissue slices in order to select the hyperintense area in the ΔUTE images.

The selected hyperintense volume, expressed as a percentage of the whole heart volume, was determined from the ΔUTE data and was correlated to the collagen rich volume as determined from Picrosirius Red stained slices in MI and control mice. To this end, a custom-built color detection algorithm based on the hue-saturation-value was used to select the whole heart from the histological images, and to select the collagen rich area (S1 Fig).

The *ex vivo* T2* signal behavior was quantified as a function of TE for two reasons: to improve our understanding of the T2*-weighted contrast for varying TEs and to determine whether the T2*-weighted signal behavior is different in post-MI and TAC hearts as compared to control hearts. To this end, average signal intensities were calculated in a stack of ten slices in the control hearts, remote tissue of the post-MI hearts, and in the TAC hearts. Signal intensities were also calculated in the selected infarct tissue of the post-MI hearts. Mean signal intensities were normalized to the signal intensity at TE = 21 μs. Next, mean signal intensity curves *SI* were fitted using a Levenberg-Marquardt least squares fitting algorithm to the model in Eq 1.

$$\text{SI}=\left|{\text{I}}_{\text{fast}}\;∙e^{-\frac{\text{TE}}{\text{T}2_{\text{fast}}^{*}}}+{\text{I}}_{\text{slow}}\;∙e^{-\frac{\text{TE}}{\text{T}2_{\text{slow}}^{*}}}+{\text{I}}_{\text{lipid}}\;∙e^{-\frac{\text{TE}}{\text{T}2_{\text{lipid}}^{*}}+i\left(\omega \;\text{TE}+\varphi \right)}\right|$$ (1)

This model consists of three exponentially decaying T2* components representing a fast relaxing pool, a slowly relaxing pool, and a lipid pool. The lipid pool decays in an oscillatory fashion due to the water-fat chemical shift difference [23]. In this equation I_fast_, I_slow_, and I_lipid_ are the relative contributions to the signal of a fast relaxing, a slowly relaxing and a lipid pool, respectively; TE is the echo time; T2*_fast_, T2*_slow_ and T2*_lipid_ the transversal relaxation times of the different pools; ω the water-lipid chemical shift difference, and *ϕ* a phase shift.

**Fig 1:**
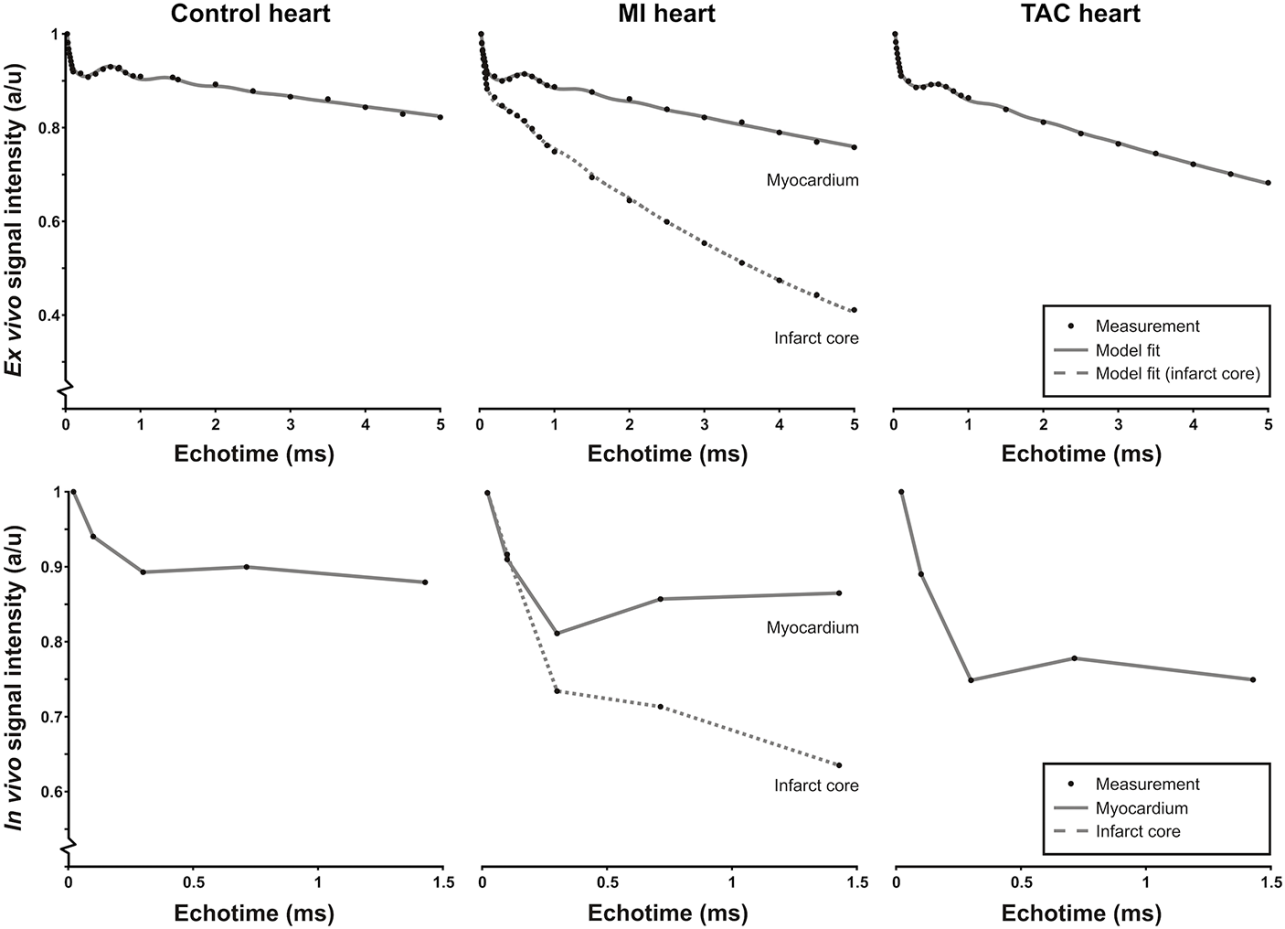
*Ex vivo* and *in vivo* signal intensity time curves. Representative ROI-based T2*-weighted signal intensity curves as a function of echo time (TE) for *ex vivo* (top row) and *in vivo* (bottom row) measurements in control hearts, remote tissue and infarct area of the post-MI hearts and the TAC hearts, together with the corresponding model fit (gray line, top row) and lines to guide the eye (bottom row). Signal intensities were normalized to the signal intensity at TE = 21 μs.

Upon inspection of the raw signal decay curves (Fig 1) it became clear that the frequency of the oscillation was approximately the same for the healthy, infarct and TAC hearts. This makes sense, since the origin of this oscillation are lipids with a fixed off-resonance frequency with respect to water. Moreover, the number of visible oscillation periods was approximately 3 in all cases, which suggested that we could fix the damping term (T2*_lipid_) of the oscillations in Eq 1 to a common value to prevent over-parameterization of the data. Therefore, based on initial model fits the water-lipid chemical shift difference was fixed to ω/2π = 1.3 kHz and T2*_lipid_ at 820 μs.

The collagen fractional area in the TAC hearts was determined from the Picrosirius Red stained slices using a custom-built color detection algorithm based on the hue-saturation-value (S2 Fig). Next, the relationship between T2*_fast_ and T2*_slow_, and the collagen fractional area of the TAC hearts was assessed.

*In vivo* long-TE images were subtracted from the corresponding images with a short-TE to obtain ΔUTE images. To quantify signal intensities a region-of-interest (ROI) analysis was performed. For the healthy and TAC hearts the LV was segmented in six slices covering the midventricular section of the heart. Average number of pixels in these LV ROIs was 932±554 and 1637±332 for the healthy and TAC hearts, respectively. For the infarct hearts, a remote tissue ROI included the basal and mid anteroseptal, basal and mid inferoseptal, and the apical septal segments (American Heart Association 17 segment model). Average number of pixels in the remote tissue ROIs was 225±97. ROIs in the infarct tissues were placed manually in the LGE hyperintense myocardium. Average number of pixels in the infarct ROIs was 141±68. To prevent any user input bias, we did not select or exclude regions based on a visual assessment of artifacts.

All T2*-weighted MR images were analyzed using home-built software in Matlab (The Mathworks, Inc.). For the cine MR images, the myocardial wall was segmented semi-automatically using the software package QMass MR (Medis, Leiden, The Netherlands) to quantify LV volumes, function and mass.

### Statistics

Data are expressed as mean±SD. Differences in LV volume, function and mass between post-MI or TAC mice were compared to control mice using a 1-sided unpaired Student’s *t*-test. Linearity of the relative Picrosirius Red-positive volume as determined with histology with the fractional volume of enhanced pixels on ΔUTE images, and of the relationship between the collagen fractional area and the T2*_fast_ and T2*_slow_ values, were assessed by performing linear regression analysis. Differences in parameters derived from the three compartment signal model between infarct and remote tissue in post-MI hearts were compared using a paired sampled Student’s *t*-test, and in remote tissue in post-MI hearts or TAC hearts and control hearts using a 2-sided unpaired Student’s *t*-test. In case the assumption of normality was violated as determined by means of a Shapiro-Wilk test, the parametric test was replaced by a Wilcoxon signed-rank test for paired or a Mann-Whitney test for unpaired data. Calculations were performed using SPSS 19.0 (SPSS Inc., Chicago). For all tests the level of significance was set at α = 0.05.

## Results

Representative end-diastolic short-axis and long-axis images of *in vivo* control, post-MI and TAC hearts are shown in S3 Fig. LV end-diastolic volume (EDV) and end-systolic volume (ESV) were increased in post-MI and TAC mice (S1 Table, *P*<0.001 in both cases). LV ejection fraction (EF) was decreased in both groups (*P<*0.001), but the cardiac output (CO) was decreased in post-MI mice only (*P*<0.05). Myocardial hypertrophy was present in post-MI and TAC mice, indicated by increased heart weight/TL and LV mass/TL (*P*<0.001 in all cases). The heart rate and respiratory rate were stable during MRI measurements, and identical in all groups.

To gain a detailed understanding of the signal formation in T2*-weighted cardiac images, we first studied the signal behavior as function of TE in excised hearts and *in vivo*. Fig 1A–1C shows representative *ex vivo* signal intensity curves as a function of TE for ROIs in control, post-MI and TAC mouse myocardium. In the *ex vivo* and *in vivo* images, the average SNR in the myocardium (TE = 21 μs) was 23±8 and 12±2, respectively. *In vivo*, typically we were only able to measure five TEs because of measurement time restrictions (Fig 1D–1F). Nevertheless, the *in vivo* curves displayed essentially the same signal decay with TE as the corresponding *ex vivo* curves, with a fast and slow decaying component and an oscillating contribution. The latter can be attributed to lipid protons for which the chemical shift difference with water protons leads to signal modulation [23]. The three components were consistently observed for the control hearts, the MI hearts and the TAC hearts. In the infarct area, the oscillating lipid signal was less prominent.

For the *ex vivo* case the signal decay as function of TE was fitted well by the 3-component model described by Eq 1 (Fig 1). Initial fits to the data resulted in an oscillation frequency of ω/2π = 1.3±0.1 kHz or 3.25±0.25 ppm, approximately corresponding to the expected water-lipid chemical shift difference. The chemical shift difference was equal for control, post-MI and TAC hearts (*P*>0.05). Also, no difference for T2*_lipid_ was observed between groups (820±470 μs, *P*>0.05). In future experiments, a fat saturation preparation sequence may be used to suppress this oscillating contribution, but for the present time we chose to fix ω and T2*_lipid_ to improve fitting accuracy of the other model parameters.

The resulting fitted relative signal contributions I_fast_, I_slow_, and I_lipid_ were almost equal between post-MI hearts and controls (Table 1). For the TAC hearts I_fast_ was slightly lower as compared to controls (*P*<0.05), whereas I_lipid_ (*P* = 0.53) and I_slow_ (*P* = 0.08) did not change. Most prominent differences between the groups were observed in the estimated T2*. T2*_slow_ in the infarct area (5.5±1.4 ms, *P*<0.001) was significantly lower as compared to the one of remote tissue, but the difference in T2*_slow_ in the remote tissue of post-MI hearts (21.0±4.3 ms) and that of control myocardium (30±11 ms) did not reach significance (*P* = 0.09). T2*_slow_ in the TAC hearts (23.0±4.7 ms) displayed a trend towards lower values as compared to controls (*P* = 0.18). T2*_fast_ was slightly increased in TAC hearts (38.0±3.9 μs) and in remote tissue of infarct hearts (50±31 μs) as compared to control hearts (34.0±3.9 μs, *P*<0.05 in both cases), and in infarct tissue (58±22 μs) as compared to remote tissue (*P*<0.05).

**Table 1:** Three-component model fit parameters of UTE signal intensity as function of TE.

	Control hearts	MI		TAC
		Remote	Infarct core	
N	6	15		13
I_fast_ (%)	14±1.8	14±2.4	11±5.4	12±1.5^*^
I_slow_ (%)	84±2.3	83±2.5	87±5.4	86±1.5
I_lipid_ (%)	2.9±0.9	2.6±1.0	1.7±1.0	2.4±0.7
T2*_fast_ (μs)	34±3.9	50±31^*^	58±22^*^	38±3.9^*^
T2*_slow_ (ms)	30±11	21±4.3	5.5±1.4^‡^	23±4.7

Listed parameters are: n = number of mice, fractions of the fast (I_fast_), slow (I_slow_) and lipid (I_lipid_) pool, and the T2*_fast_ (μs) and T2*_slow_ (ms) for control hearts, infarct and remote tissue in post-MI hearts, and the TAC hearts. Indicated are:

* (*P*<0.05)

† (*P*<0.01)

‡(*P*<0.001) of the TAC hearts or the remote tissue in post-MI hearts as compared to the control hearts, or between the infarct core and remote tissue in post-MI hearts. The change of T2*_slow_ in remote tissue of post-MI hearts, as compared to control hearts, did not reach statistical significance (*P* = 0.09, respectively), so as the change of T2*_slow_ in TAC hearts as compared to control hearts (*P* = 0.18).

**Fig 2:**
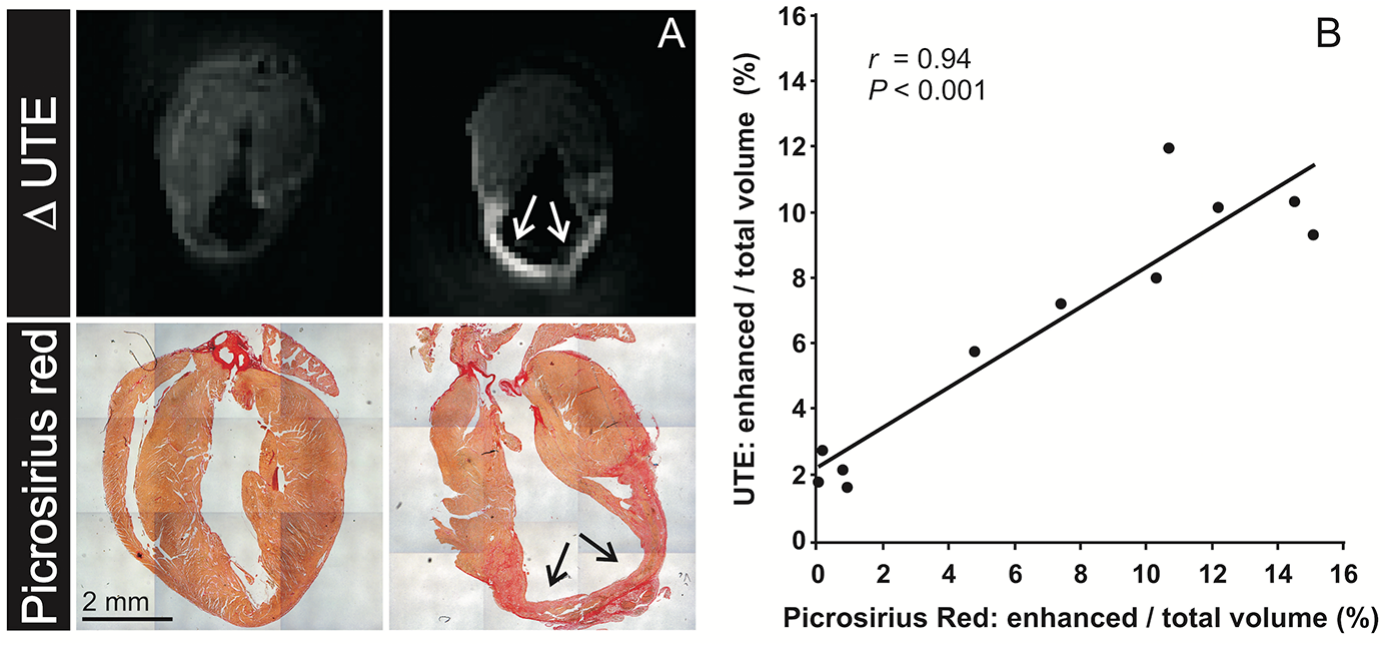
Relative infarct size determined from histology compared to MRI. (A) Long axis cross sections through *ex vivo* ΔUTE images in post-MI mouse hearts obtained from subtraction of a long-TE (4 ms) from a short-TE (21 μs) T2*-weighted image. The left panels show a post-MI heart 2 days after surgery. The right panels show a post-MI heart with a chronic MI 7 days after surgery. A positive contrast is observed in the ΔUTE image, which corresponds to the location of the chronic MI. Corresponding Picrosirius red stained slices showed hardly any collagen in the MI heart 2 days after surgery, whereas excessive replacement fibrosis was present (arrows) in the chronic MI. (B) Correlation between the infarct size as percentage of the total heart volume determined from histology and the *ex vivo* ΔUTE images of control (n = 3) and MI hearts (n = 8). The solid line is a linear fit.

Having established that the most prominent signal difference between infarct and remote tissue concerns the T2*_slow_, we exploited this finding to obtain image contrast on long- and short-TE subtraction images (referred to as ΔUTE images), which is a common approach to improve contrast and suppress background signal. A representative *ex vivo* ΔUTE image of a chronic post-MI heart with a considerable fibrotic scar in the apical region is shown in Fig 2A. The image is shown next to a MI heart 2 days after surgery with substantial wall thinning but without a collagenous scar. A clear hyperenhancement in the ΔUTE image was observed in the apex of the chronic post-MI heart, which co-localized with the area of fibrous scarring observed by histology. The relative infarct volume (% of the whole heart volume) as determined from the ΔUTE images correlated linearly with infarct volume from histology (r = 0.94, *P*<0.001) (Fig 2B). T2* is often associated with the presence of iron in tissue. However, hardly any iron was visible on Prussian blue stained tissue sections (S4 Fig), ruling out iron as the source of T2* decrease.

**Fig 3:**
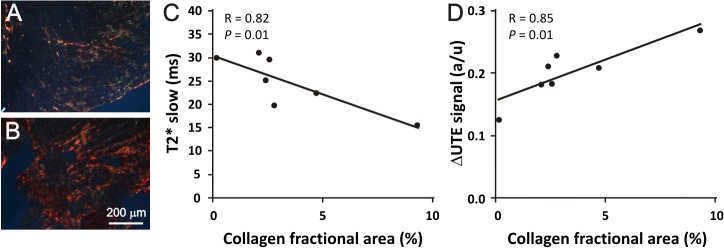
Collagen fraction area determined from histology compared to T2*slow and the ΔUTE signal. Representative picrosirius red stained slice of a TAC heart with (A) low amounts and (B) of a heart with high amounts of collagen present. Correlations between (C) T2*_slow_ and (D) ΔUTE contrast versus the collagen fractional area of control (n = 1) and TAC hearts (n = 6). The solid lines are linear fits.

*Ex vivo* analysis (Fig 3) revealed a moderate, but significant, linear correlation of T2*_slow_ as well as ΔUTE image intensity with the amount of diffuse fibrosis in the TAC hearts (r = 0.82, *P* = 0.01 and r = 0.85, *P* = 0.01, respectively). This demonstrates that T2* reported on the presence of diffuse fibrosis in these hearts.

**Fig 4:**
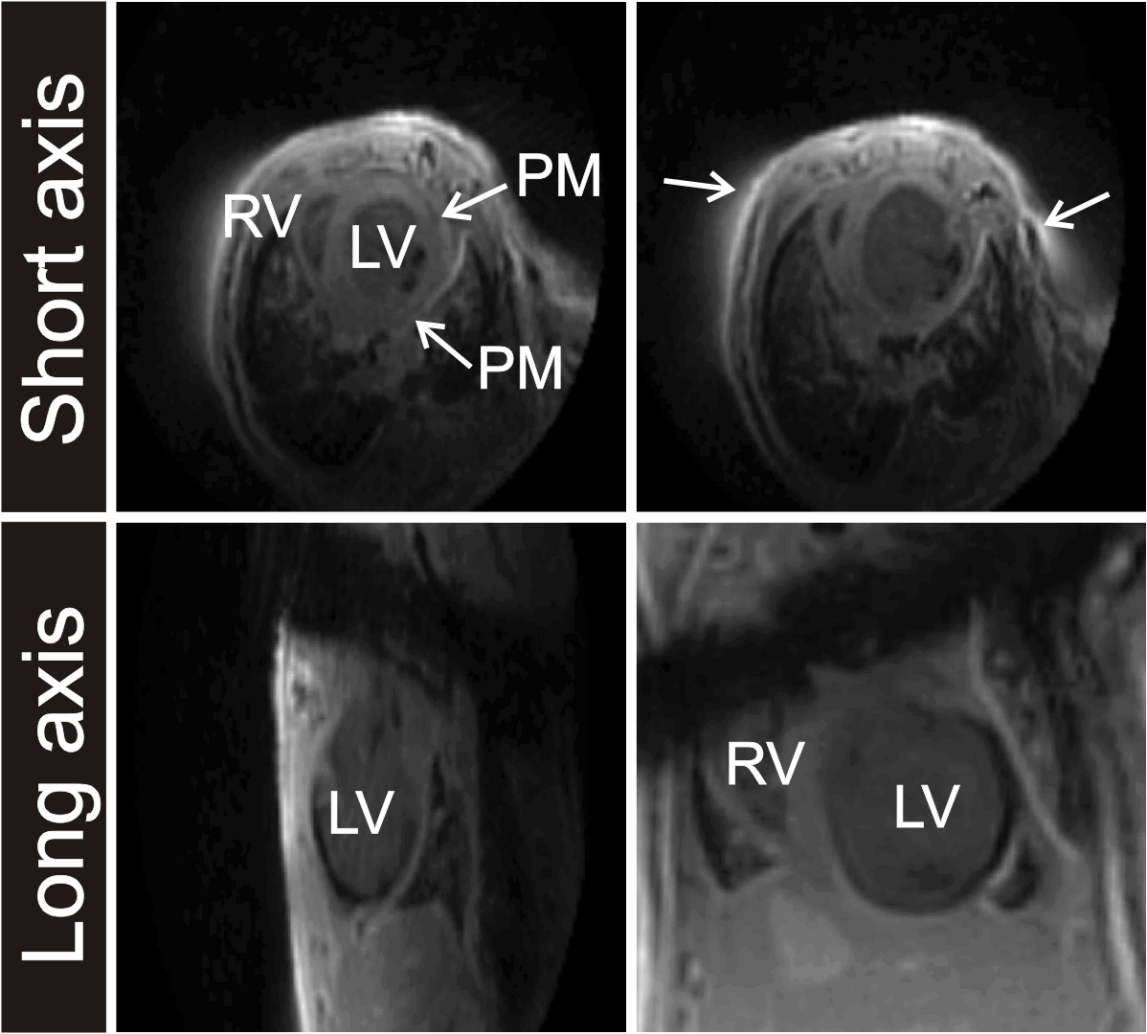
T2*-weighted MR images of a post-MI mouse heart. Two short-axis and long-axis cross-sections through an *in vivo* T2*-weighted dataset (TE = 21 μs) of a post-MI mouse heart. Indicated are the right ventricle (RV), left ventricle (LV), the papillary muscles (PM) and some small artefacts (↖). A dark saturation band with low signal intensity is visible in the two long-axis images.

*In vivo* imaging of replacement and diffuse fibrosis in the mouse models was performed with cardiac triggered versions of the T2*-weighted sequence. The resulting basic image quality was adequate, as demonstrated for a post-MI mouse heart (Fig 4). Left and right ventricular wall, papillary muscles and infarct area, as well as the surrounding anatomy of ribs and lungs could be clearly distinguished. The dark bands in the long-axis images resulted from the saturation slice to suppress blood signal in the LV lumen.

**Fig 5:**
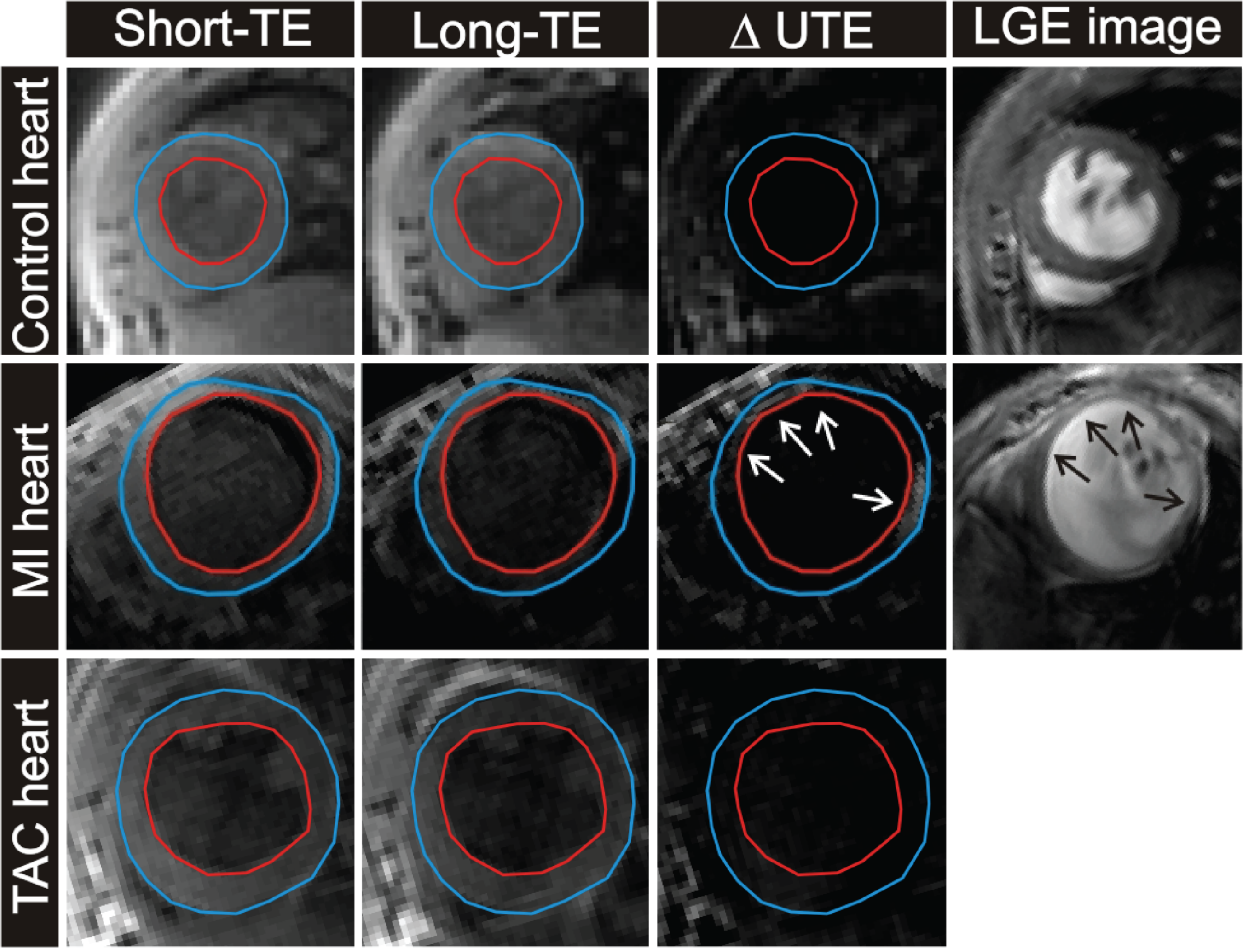
*In vivo* T2*-weighted images in control heart, a MI heart and a TAC heart. Examples of a short-axis cross-section of a control heart (top row), a MI heart (middle row) and a TAC heart (bottom row) obtained with the T2*-weighted 3D sequence (short-TE, long-TE and ΔUTE image). LGE scans were obtained in control and post-MI mice (right column). The arrows indicate the infarcted areas.

**Fig 6:**
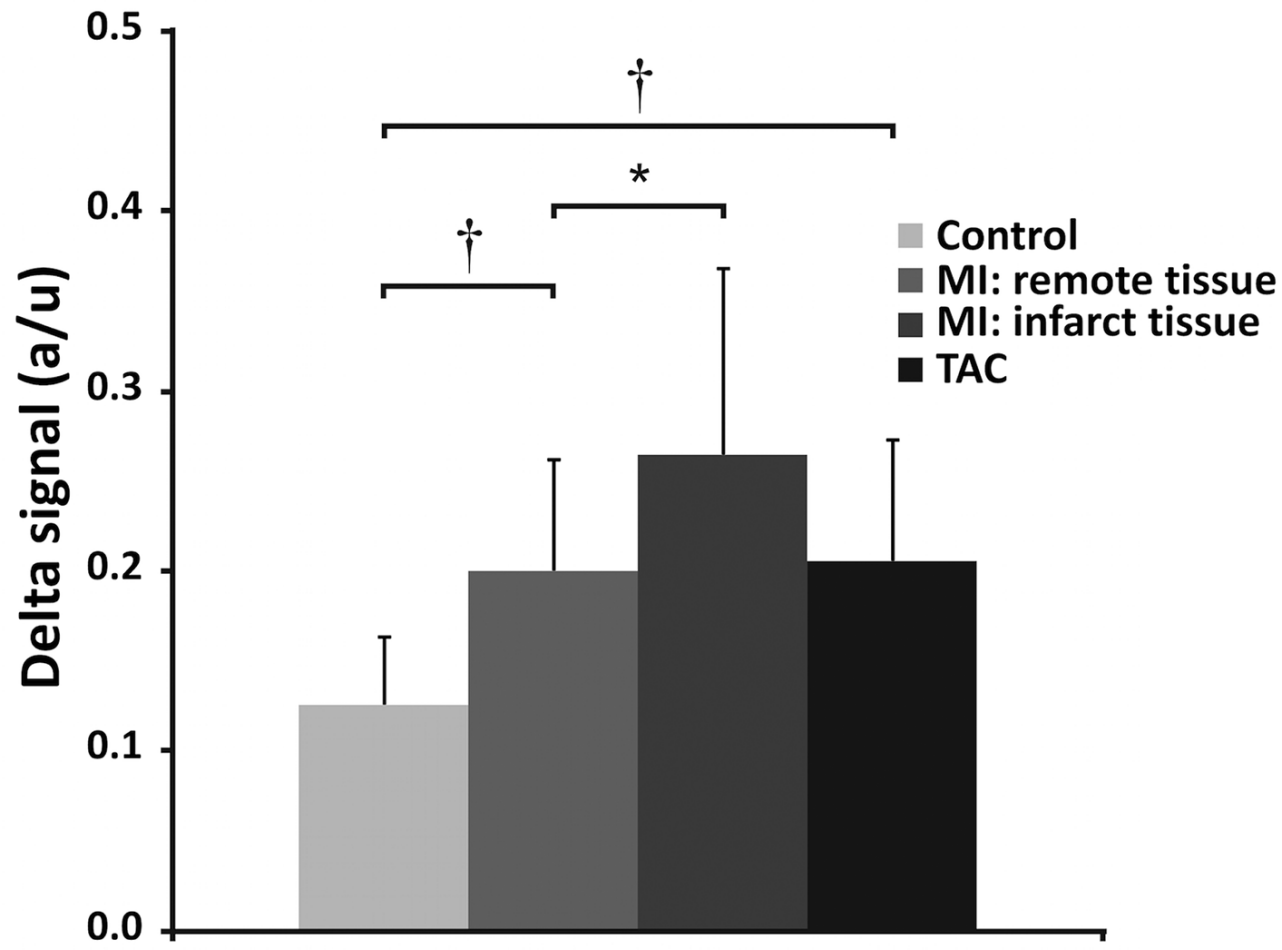
*In vivo* signal difference between long and short-TE. *In vivo* signal difference between long and short-TE, in control, post-MI and TAC mouse hearts. The signal difference between the short-TE (21 μs) and long-TE images (1.429 ms) is larger for remote and infarct tissue in post-MI hearts, and in TAC hearts, compared to control hearts. * (*P*<0.05) and † (*P*<0.01). Error bars indicate SD.

Direct visual assessment of the fibrosis in the *in vivo* ΔUTE images, however, proved more challenging (Fig 5). ΔUTE images of the post-MI hearts revealed areas with hyperenhancement, which co-localized with the MI as independently determined by LGE. However, ΔUTE contrast was significantly less pronounced compared to the *ex vivo* measurements and in many cases difficult to distinguish visually. As expected for diffuse fibrosis, in the TAC hearts no focal hyperenhancement was observed (Fig 5). Nevertheless, an ROI-based ΔUTE signal differences of control, post-MI and TAC mice (Fig 6) demonstrated that ΔUTE signal in the MI (0.26±0.10) was significantly higher than that in remote tissue (0.20±0.06, *P<*0.05). Moreover, the ΔUTE signal in the remote tissue of the post-MI hearts (0.20±0.06) and that in the TAC hearts (0.21±0.07) was larger as compared to that in the control hearts (0.13±0.04, *P<*0.001 vs. post-MI, and *P<*0.001 vs. TAC). We attribute the latter signal difference to the presence of diffuse fibrosis in these hearts, in line with the *ex vivo* results of Fig 3.

## Discussion

Myocardial fibrosis has an important impact on heart disease progression and is related to poor outcome [4,24]. Replacement and diffuse fibrosis may be visualized using a Gd-chelate contrast agent and T1-weighted imaging or quantitative T1-mapping [25,26]. However, the use of gadolinium contrast is contraindicated for a large group of patients with poor kidney function [27]. Alternative imaging techniques for the detection of replacement and, more importantly, diffuse fibrosis are therefore highly desired for this patient group. Here, we have explored the use of T2* to image replacement and diffuse fibrosis by virtue of changes in this relaxation time parameter induced by the presence of collagen and other fibrotic elements in the ECM.

Detailed T2* measurements with varying TE were performed in excised hearts, to improve the understanding of the T2*-weighted signal decay and to investigate whether signal behavior is different in case of replacement or diffuse fibrosis. Typically, transverse relaxation processes in muscle tissue are best described by a multi-component signal model depending on the range of TEs used [28]. Indeed, the T2* signal decay curves were adequately described by two exponentially decreasing and one oscillating decaying lipid component. The relative contributions of the model components (I_fast_, I_slow_, and I_lipid_) were essentially the same in control, post-MI and TAC hearts, apart from a small decrease of I_fast_ in the TAC hearts as compared to controls (Table 1). This is a surprising finding, since there are rather large differences in the amounts of collagen and other extracellular matrix components in the myocardium for the different models, particularly for the MI tissue in comparison to remote and control tissue, which were not reflected in the relative contributions of the model components. We propose two potential reasons for this finding. First, the signal was normalized to the shortest achievable TE (21 μs), which may not be sufficiently short to capture the entire signal from collagen-bound water. Possible differences in signal behavior at even shorter TE may therefore have gone undetected. Second, different T2* contributions are subject to partial volume effects, since most of the MRI signal in the myocardium originates from free water that undergoes rapid exchange between the extracellular and intracellular tissue compartments. In this respect, it is not unlikely that changes in the relaxation behavior mediated by changes in collagen content were detected in the water pool with the highest signal contribution and the longer transverse relaxation time. The significant correlation between the collagen fractional area and the T2*_slow_ as determined in *ex vivo* TAC hearts supports this explanation, as well as the shorter T2*_slow_ and the longer T2*_fast_ in *ex vivo* MI and TAC hearts.

Previously, strong correlations were reported between the amount of diffuse fibrosis and T2 in a small animal model of diabetic cardiomyopathy [12]. Our findings are also in quantitative agreement with recent measurements by Aguor *et al*. who found a significant decrease in T2* in MI using a multi-gradient-echo sequence [29].

Hyperenhancement in ΔUTE images colocalized with collagen rich areas in MI tissue observed in stained sections, and the hyperintense volume from MRI and enhanced volume from histology strongly correlated. Iron can be ruled out as a significant source of contrast in this study as hardly any iron was detected in Prussian blue stained slices of the MI tissue (S4 Fig) [30], although iron deposits in reperfused myocardium will hinder unambiguous interpretation of T2*-weighted images [31]. These findings are in agreement with the *ex vivo* results of de Jong *et al*. who also reported a clear correlation between relative enhanced volume from UTE MRI and MI size from histology for a rat MI model [13].

Nevertheless, *in vivo* signal enhancement in the ΔUTE images was less convincing (Fig 5). We believe that partial volume effects in the thin infarct myocardium may have obscured some of the contrast. Also, despite cardiac triggering, there might have been some misalignment due to residual cardiac motion. Improvements in the imaging sequence towards higher resolution in shorter scan times are needed to resolve these issues.

It would be preferable to perform *in vivo* T2* mapping, since this would facilitate quantitative comparisons between studies or in longitudinal studies and might strongly reduce the sensitivity to sequences parameters, coil sensitivity profiles, and cardiac and respiratory rates. As a drawback, accurate T2* quantification relies on magnetic field homogeneity. Currently, the acquisition time of the T2*-weighted MR measurements is too long to enable *in vivo* T2* mapping, for which acquisition acceleration techniques such as parallel imaging or compressed sensing are a necessity [32].

Complementary techniques for fibrosis imaging include the use of collagen-targeted MRI contrast agents [33]. Also, fibrosis-induced changes in tissue stiffness may be quantified using cardiac MR elastography [34] or by detailed evaluation of LV diastolic function [24,26]. Alternatively, ultrasonic reflectivity was shown to relate to the amount of connective tissue in human myocardium [35].

Complications in the present experimental design include age differences between the MI and TAC mice. However, changes in the ECM composition due to ageing are known to be much smaller than fibrosis by MI or TAC [36]. Furthermore, differences in blood oxygenation, relative blood volume and edema are known to modulate tissue T2* properties and could therefore affect ΔUTE contrast and T2* values [37–39]. We can exclude significant effects of edema, since this mainly occurs in the acute phase after cardiac ischemia and would lead to longer transversal relaxation times in the MI tissue [40]. The blood oxygenation level might also influence the myocardial transverse relaxation time [41]. We cannot fully exclude such a contribution to the observed contrast, because we have no information on the oxygenation of the mouse blood. However, the strong contrast in the MI tissue observed *in vivo* and *ex vivo*, where little blood is present, and the strong correlation between T2* and the amount of interstitial collagen in the TAC model observed on histology strongly suggest that fibrosis is the main source of contrast.

## Summary

We have investigated imaging of fibrosis in the myocardium by quantification of fibrosis-induced shortening of T2*. T2* decay in the myocardium was multi-exponential. In mice post MI, the infarct area was characterized by a more rapid T2* decay as compared to remote tissue. Short and long-TE subtraction images depicted a positive contrast in the infarct, which co-localized with the fibrotic scar and quantitatively correlated with infarct volumes observed on histology. Although less pronounced, T2* decay was also faster in the presence of diffuse fibrosis in the myocardium of TAC mice. T2* values and contrast in short and long-TE subtraction images correlated with the amount of diffuse fibrosis. Fibrosis-induced T2* lowering in post-MI and TAC mice was also observed *in vivo*. However, *in vivo* contrast on subtraction images was rather poor, hampering a straightforward visual assessment of the spatial distribution of the fibrotic tissue.

## Supporting Information

S1 Fig Infarct size quantification in MI mice from histology. (A) Representative stack of ten Picrosirius stained slices of a post-MI heart. A custom-built color detection algorithm was used to define (B) a whole heart mask to remove the background from (C) the histological images. To this end, the original RGB images (red-green-blue) were converted to HSV images (hue-saturation-value) and signal intensity thresholds were applied to the different HSV channels. (D) Next, a mask was defined to select (E) the collagen rich area, using a signal intensity threshold on the hue channel. The HSV thresholds were determined empirically and proper selection of the heart and collagen rich area were confirmed by visual inspection.
(PDF)

S2 Fig Collagen fractional area in TAC mice determined from histology. Representative stack of ten Picrosirius stained slices of a TAC heart (A). A custom-built color detection algorithm was used to define a mask (B) removing the background from the histological images (C). To this end, the original RGB images (red-green-blue) were converted to HSV images (hue-saturation-value) and signal intensity thresholds were applied to the different HSV channels. Next, a mask (D) was defined to select the collagen (E). The HSV thresholds were determined empirically and proper selection of the heart and collagen rich area were confirmed by inspection.
(PDF)

S3 Fig Cinematographic MRI. Representative end diastolic short-axis and long-axis images from a control heart, a post-MI heart and a TAC heart.
(PDF)

S4 Fig Prussian blue staining. Representative Prussian Blue stained slices of (A) a control heart and (B) the infarct area of a post-MI heart. Hardly any iron deposits were present in the healthy hearts and only very small amounts were found in the infarct area (↖).
(PDF)

S1 Methods Procedure for animal surgery and histology. (PDF)

S1 Table General animal characteristics and global LV cardiac parameters determined from cine MRI. (PDF)
