# Supplementary material for: Assessment of Myocardial Fibrosis in Mice Using a T2*-Weighted 3D Radial Magnetic Resonance Imaging Sequence
Source: PLoS One. 2015 Jun 26;10(6):e0129899. doi: 10.1371/journal.pone.0129899 (PMC4482648; doi:10.1371/journal.pone.0129899)
Supplement: S1 Fig — (A) Representative stack of ten Picrosirius stained slices of a post-MI heart. A custom-built color detection algorithm was used to define (B) a whole heart mask to remove the background from (C) the histological images. To this end, the original RGB images (red-green-blue) were converted to HSV images (hue-saturation-value) and signal intensity thresholds were applied to the different HSV channels. (D) Next, a mask was defined to select (E) the collagen rich area, using a signal intensity threshold on the hue channel. The HSV thresholds were determined empirically and proper selection of the heart and collagen rich area were confirmed by visual inspection. (PDF) [file pone.0129899.s001.pdf]

Supplemental information to:

Assessment of myocardial fibrosis in mice using a T2*-weighted 3D radial magnetic resonance imaging sequence

Bastiaan J. van Nierop, Noortje A.M. Bax, Jules L. Nelissen, Fatih Arslan, Abdallah G. Motaal, Larry de Graaf, Jaco J.M. Zwanenburg, Peter R. Luijten, Klaas Nicolay, Gustav J. Strijkers

Supplemental Figure 1: Infarct size quantification from histology.

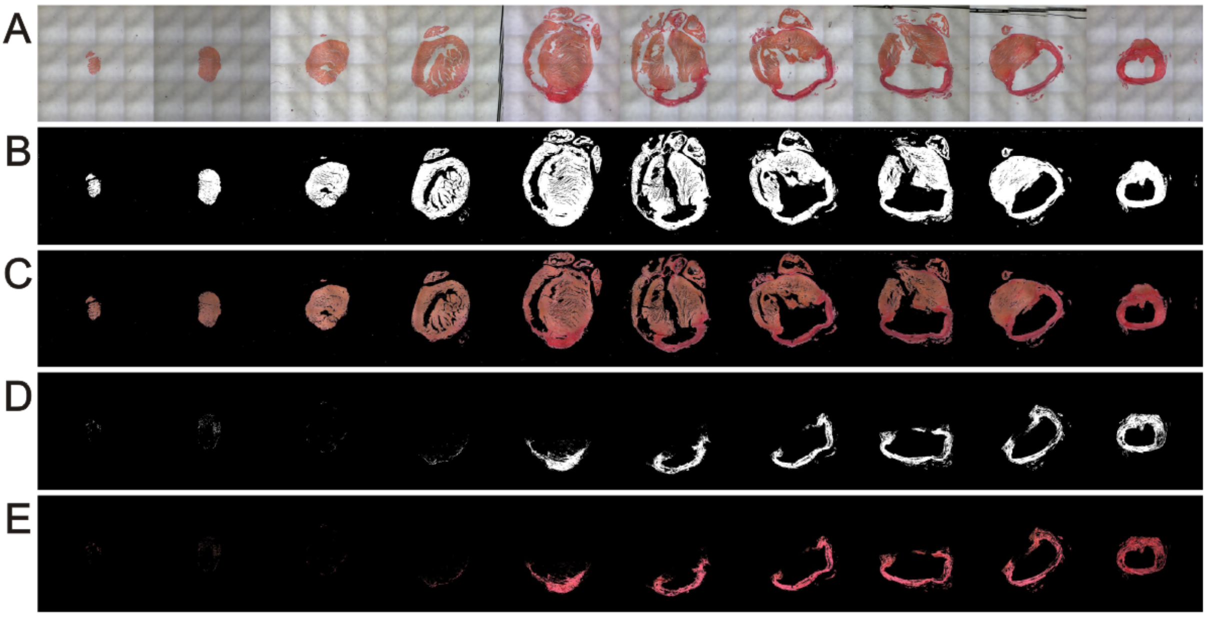

(A) Representative stack of ten Picrosirius stained slices of a post-MI heart. A custom-built color detection algorithm was used to define (B) a whole heart mask to remove the background from (C) the histological images. To this end, the original RGB images (red-green-blue) were converted to HSV images (hue-saturation-value) and signal intensity thresholds were applied to the different HSV channels. (D) Next, a mask was defined to select (E) the collagen rich area, using a signal intensity threshold on the hue channel. The HSV thresholds were determined empirically and proper selection of the heart and collagen rich area were confirmed by visual inspection.
