# Supplementary material for: Assessment of Myocardial Fibrosis in Mice Using a T2*-Weighted 3D Radial Magnetic Resonance Imaging Sequence
Source: PLoS One. 2015 Jun 26;10(6):e0129899. doi: 10.1371/journal.pone.0129899 (PMC4482648; doi:10.1371/journal.pone.0129899)
Supplement: S3 Fig — Representative end diastolic short-axis and long-axis images from a control heart, a post-MI heart and a TAC heart. (PDF) [file pone.0129899.s003.pdf]

Supplemental information to:
Assessment of myocardial fibrosis in mice using a T2*-weighted 3D radial magnetic resonance imaging sequence

Bastiaan J. van Nierop, Noortje A.M. Bax, Jules L. Nelissen, Fatih Arslan, Abdallah G. Motaal, Larry de Graaf, Jaco J.M. Zwanenburg, Peter R. Luijten, Klaas Nicolay, Gustav J. Strijkers

Supplemental Figure 3: Cinematographic MRI.

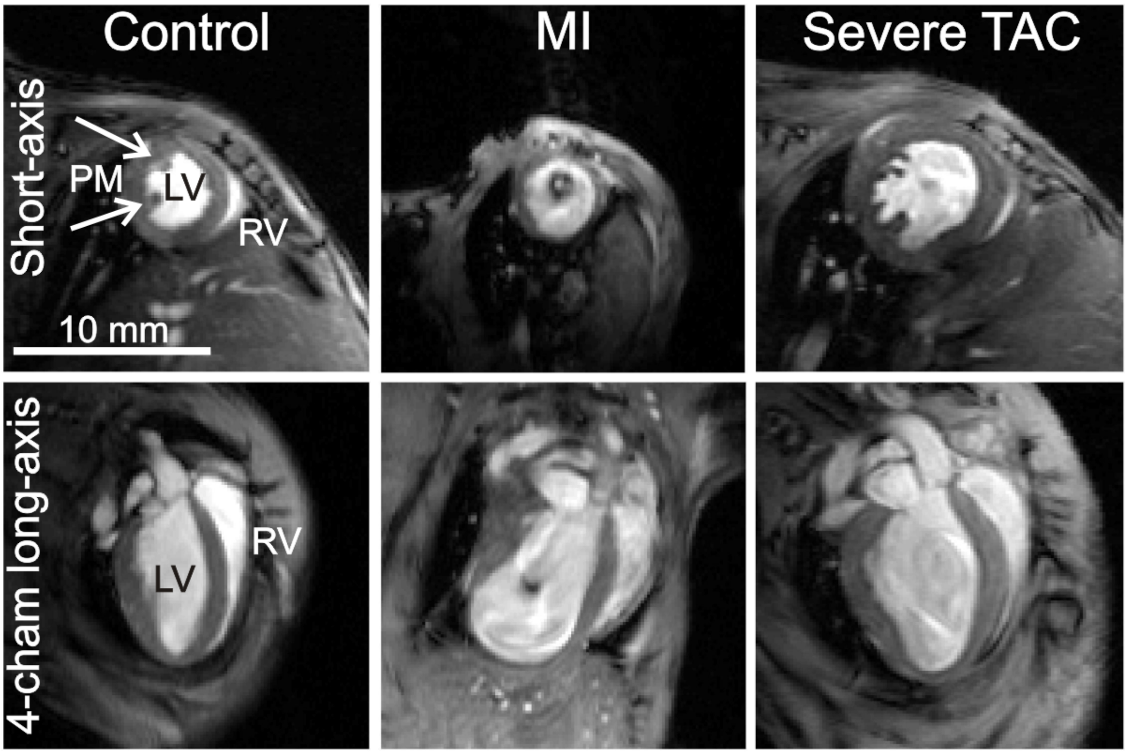

Representative end diastolic short-axis and long-axis images from a control heart, a post-MI heart and a TAC heart.
