# Supplementary material for: Assessment of Myocardial Fibrosis in Mice Using a T2*-Weighted 3D Radial Magnetic Resonance Imaging Sequence
Source: PLoS One. 2015 Jun 26;10(6):e0129899. doi: 10.1371/journal.pone.0129899 (PMC4482648; doi:10.1371/journal.pone.0129899)
Supplement: S4 Fig — Representative Prussian Blue stained slices of (A) a control heart and (B) the infarct area of a post-MI heart. Hardly any iron deposits were present in the healthy hearts and only very small amounts were found in the infarct area (↖). (PDF) [file pone.0129899.s004.pdf]

Supplemental information to:

Assessment of myocardial fibrosis in mice using a T2*-weighted 3D radial magnetic resonance imaging sequence

Bastiaan J. van Nierop, Noortje A.M. Bax, Jules L. Nelissen, Fatih Arslan, Abdallah G. Motaal, Larry de Graaf, Jaco J.M. Zwanenburg, Peter R. Luijten, Klaas Nicolay, Gustav J. Strijkers

Supplemental Figure 4: Prussian blue staining.

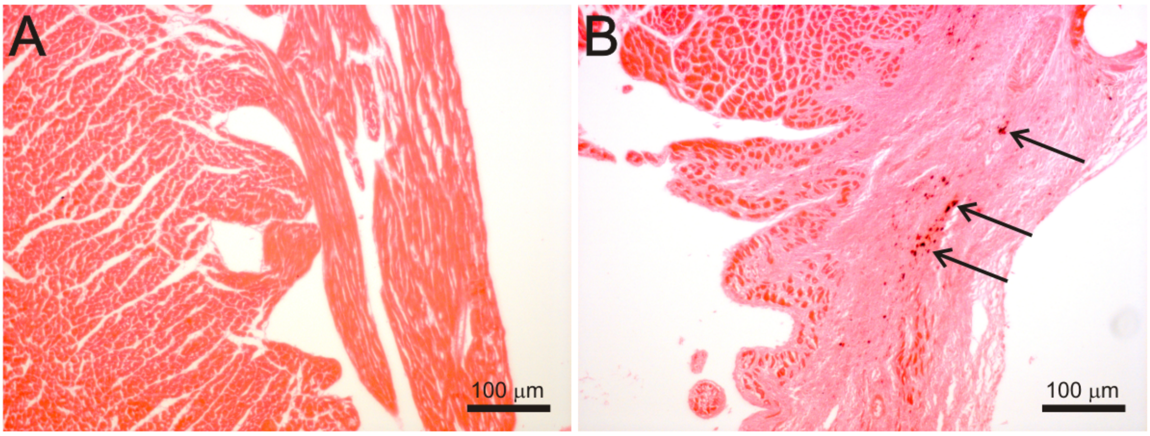

Representative Prussian Blue stained slices of (A) a control heart and (B) the infarct area of a post-MI heart. Hardly any iron deposits were present in the healthy hearts and only very small amounts were found in the infarct area (%).
