# Supplementary material for: Assessment of Myocardial Fibrosis in Mice Using a T2*-Weighted 3D Radial Magnetic Resonance Imaging Sequence
Source: PLoS One. 2015 Jun 26;10(6):e0129899. doi: 10.1371/journal.pone.0129899 (PMC4482648; doi:10.1371/journal.pone.0129899)
Supplement: S1 Methods — (PDF) [file pone.0129899.s005.pdf]

Supplemental information to:

Assessment of myocardial fibrosis in mice using a T2*-weighted 3D radial magnetic resonance imaging sequence

Bastiaan J. van Nierop, Noortje A.M. Bax, Jules L. Nelissen, Fatih Arslan, Abdallah G. Motaal, Larry de Graaf, Jaco J.M. Zwanenburg, Peter R. Luijten, Klaas Nicolay, Gustav J. Strijkers

Supplemental methods

Procedure for animal surgery

Mice were anesthetized with 2.5 vol% isoflurane (Abbott Laboratories Ltd, Maidenhead, Berkshire, UK) in 0.2 L/min O_2 and 0.2 L/min medical air and intubated for mechanical ventilation. Animals were placed on a heating pad to maintain body temperature at 37 °C. Buprenorphine (0.1 mg/kg s.c.) (Schering-Plough BV., Houten, The Netherlands) was administered for analgesia. Surgical procedures were performed using a stereo microscope (Leica M80). To induce MI the following steps were taken. Mice were placed in a supine position. The left thorax was opened in the fourth intercostal space, the pericardium was opened and the LAD was tied off (6-0 silk suture) just behind its main bifurcation. The success of the procedure was confirmed by discoloration of the myocardium. Finally, the thorax was closed. To induce a stenosis of the aorta in the TAC model a small incision was made just lateral from the sternum above the first intercostal space. The aortic arch was exposed and tied off (6-0 silk suture) together with a 27G (Ø 0.42 mm) needle between the innominate artery and the left common carotid artery. Thereafter, the needle was immediately removed, restoring blood flow. Then, the chest was closed. All surgically prepared animals were allowed to recover at 30 ^{o}C.

Histology

A subset of the healthy (n=4), MI (n=8) and TAC (n=6) hearts were embedded in paraffin and cut in 5-µm-thick sections, as described previously[1]. Serial sections (50 µm interval) of the healthy and MI hearts were stained with Picrosirius Red and with Prussian Blue according to standard histological

---

[1] Winter EM et al. *Circulation*. 2007;116:917-927

procedures to confirm the excessive presence of collagen in the infarct area and to exclude confounding effects of iron deposits on the signal formation in the *ex vivo* UTE images, respectively. Serial sections (100 µm interval) covering a one mm mid ventricular portion of the TAC hearts were stained with Picrosirius Red to quantify the collagen fractional area in these hearts, defined as the collagen surface area as percentage of the total myocardial surface area. The stained sections were digitalized with a Zeiss Axio Observer Z1 microscope equipped with an AxioCam MRc5 digital camera. Conventional transmission microscopy was used for the healthy and post-MI hearts yielding a total of approximately 38 images per heart. Polarization microscopy was used for quantification of collagen fractional area in the TAC hearts yielding a total of approximately 58 images per heart.
