# Supplementary material for: Assessment of Myocardial Fibrosis in Mice Using a T2*-Weighted 3D Radial Magnetic Resonance Imaging Sequence
Source: PLoS One. 2015 Jun 26;10(6):e0129899. doi: 10.1371/journal.pone.0129899 (PMC4482648; doi:10.1371/journal.pone.0129899)
Supplement: S1 Table — (PDF) [file pone.0129899.s006.pdf]

Supplemental information to:

Assessment of myocardial fibrosis in mice using a T2*-weighted 3D radial magnetic resonance imaging sequence

Bastiaan J. van Nierop, Noortje A.M. Bax, Jules L. Nelissen, Fatih Arslan, Abdallah G. Motaal, Larry de Graaf, Jaco J.M. Zwanenburg, Peter R. Luijten, Klaas Nicolay, Gustav J. Strijkers

Supplemental Table 1: General animal characteristics and global LV cardiac parameters determined from cine MRI of the cohort of control mice, post-MI mice and TAC mice.

	Control			MI			TAC		
n	10			18			12		
HW [mg]	156	±	17	181	±	29*	252	±	72‡
HW/TL [mg/mm]	8.7	±	0.6	10.1	±	1.6*	13.9	±	4.0‡
LV mass [mg]	85	±	9	109	±	22‡	135	±	33‡
LV mass/TL [mg/mm]	4.7	±	0.5	6.3	±	1.1‡	7.6	±	2.0‡
LuW [mg]	179	±	35	157	±	23	213	±	75
LuW/TL [mg/mm]	10.4	±	2.1	8.8	±	1.2	11.7	±	4.2
HR [min^{-1}]	509	±	33	519	±	42	518	±	46
Resp [min^{-1}]	81	±	12	79	±	10	83	±	9
EF [%]	61	±	7	33	±	14‡	42	±	18‡
EDV [µl]	68	±	11	125	±	47‡	108	±	38‡
ESV [µl]	27	±	7	89	±	48‡	68	±	42‡
SV [µl]	41	±	7	36	±	10	40	±	10
CO [ml min^{-1}]	22	±	3	19	±	6*	21	±	5

Listed parameters are: n = number of mice, HW = heart weight, HW/TL = heart weight normalized to tibia length, LV mass = left ventricular myocardial mass as determined using MRI, LV mass/TL is LV mass normalized to tibia length, LuW = lung weight, LuW/TL = lung weight normalized to tibia length, HR = heart rate, Resp = respiratory rate, EF = ejection fraction, EDV = end diastolic volume, ESV = end systolic volume, SV = stroke volume, CO = cardiac output. Statistical differences are indicated by * ($P<0.05$), † ($P<0.01$) and ‡ ($P<0.001$).
